# The abrupt transition from face-to-face to online treatment for eating disorders: a pilot examination of patients’ perspectives during the COVID-19 lockdown

**DOI:** 10.1186/s40337-021-00383-y

**Published:** 2021-03-05

**Authors:** Yael Doreen Lewis, Roni Elran-Barak, Rinat Grundman-Shem Tov, Eynat Zubery

**Affiliations:** 1 Hadarim Eating Disorders Treatment Center, Shalvata Mental Health Center, Hod Hasharon, Israel; 2 Sackler Faculty of Medicine, Tel Aviv University, Tel Aviv, Israel; 3 School of Public Health, University of Haifa, Haifa, Israel

**Keywords:** Eating disorders, COVID-19, Coronavirus, Lockdown, Telemedicine, Telehealth, eHealth, Metal health

## Abstract

**Background:**

Studies investigating patients’ perspectives towards an abrupt transition from face-to-face to online treatment in eating disorders (EDs) are scarce. The current study aimed to (1) conduct a preliminary assessment of patients’ perspectives regarding this transition, and (2) explore potential demographic, clinical, and treatment-related factors associated with these perspectives.

**Methods:**

Sixty-three patients with EDs whose treatment was moved to an online format, were surveyed during the COVID-19 lockdown (April–May 2020). A 6-item measure was developed to examine their perspectives toward this transition. Exploratory factor analyses (EFAs) were conducted to confirm the rational-theoretical structure of the measure (Eigenvalue = 3.745, explaining 62.4% of variance). The Cronbach’s alpha value was excellent (α = 0.878). Validated questionnaires were used to measure ED symptoms, general psychopathology, therapeutic alliance, and pandemic anxiety, and their associations with our transition-focused scale and telemedicine satisfaction were examined.

**Results:**

Mixed views were found regarding the transition, with the majority (68%) stating that they would not choose to continue online therapy given the option. Longer duration of treatment (r = 0.291, *p* = 0.022), stronger therapeutic alliance (r = 0.293, *p* = 0.028), and higher COVID-19 anxiety (r = 0.276, *p* = 0.029) were linked with more positive views towards the transition.

**Conclusions:**

Analyses suggest that patients’ perspectives towards the transition can be measured using a Likert-type 6-item scale. Findings highlight the various responses to online treatment and indicate a need to identify patients who may face difficulties in the transition to this newly ubiquitous treatment mode. Clinicians should be cognizant of these potential difficulties and consider appropriate modes of treatment in the ongoing pandemic situation.

## Plain English summary

The topic of forced and abrupt changes of setting in psychological treatment was, until the COVID-19 pandemic struck, scarcely ever addressed. Despite the obligatory massive shift to online treatment because of worldwide lockdowns, little was previously known about patients’ attitudes towards such a transition and/or possible outcomes. The aim of the current study was to conduct a preliminary assessment of patients’ perspectives regarding the transition to online therapy during the first COVID-19 lockdown, and explore potential factors associated with these perspectives. We found that longer duration of pre-lockdown treatment, stronger therapeutic alliance, and high COVID-19 anxiety were associated with a more positive perspective toward the transition. These findings could inform further research on similar transitions, as well as drawing clinicians’ attention to the various implications of such transitions.

## Background

The subject of obligatory changes in the setting of psychological treatment has received little attention in the scientific literature. Efforts to control the spread of the novel severe acute respiratory syndrome coronavirus 2 (SARS-CoV-2) have led to quarantining and social distancing, across the globe, accompanied by considerable psychological implications [1, 2]. In this context, ambulatory mental health services shifted from conventional face-to-face treatment to remote online treatment. Although some preliminary reports have emerged regarding therapists’ attitudes [3], patients’ experiences of this abrupt transition have received little attention [4].

Online mental health treatment, including psychological and psychiatric services, has been found to be effective in both the assessment and delivery of care across diagnoses [5, 6], including eating disorders (EDs) [7], although more research is warranted to understand benefits and possible adverse effects. During the coronavirus disease (COVID-19) lockdown, patients with EDs experienced altered access to services [8]. A rapid shift from the previous limited scale of tele-mental health care, to its wide usage, was thus urged worldwide [9–11], including in the area of EDs [12]. Taking into account predictions for an increase in ED risks and symptoms during the COVID-19 crisis via several pathways related to disrupted food access and routines, movement and exercise constraints, heightened stress and anxiety, decreased support and, in some cases, the increased possibility of violence [13, 14], the provision of continuous therapy remained a principal goal. However, this unprecedented and abrupt transition was forced upon patients as well as on therapists, with no prior warning or preparation. For guidance, and due to the lack of studies describing patients’ perspectives, therapists were suddenly in a position of having to rely on the limited literature [15], on in-house institutional professional peer support [16], and on an international exchange of viewpoints and expertise [17].

In the absence of existing knowledge about the patient experience concerning such an abrupt change, we wished to conduct a pilot study investigating how patients in ambulatory treatment for EDs perceived the transition from traditional face-to-face treatment to remote online treatment. We sought to study a unique “real-world setting” situation created by the imposition of the first lockdown, in contrast to previous studies of telehealth in EDs in which participants had a choice of treatment. Therefore, our aims were to (1) assess patients’ perspectives regarding this transition, and (2) explore potential demographic, clinical, and treatment-related factors associated with these perspectives. Specifically, we chose to consider the factors of depression and anxiety symptoms, which are significant comorbidities and have been suggested to be linked with EDs [18], as well as COVID-19 anxiety. With respect to therapy-related factors, it seemed crucial to investigate the therapeutic alliance, which has been shown to be related to patients’ perceptions of their therapy outcome [19]. Patient satisfaction with online treatment was also measured in order to draw a clear distinction between patients’ perceptions towards the transition to online treatment and their satisfaction with it.

Considering that the initial phase of therapy is a critical period for the formation of the therapeutic alliance, necessary for the building of trust and collaborative work [20], and in light of the unexpected challenge of building an alliance remotely [21], we hypothesized that patients with established therapeutic relationships (i.e., those who had been in face-to-face therapy for a longer time before the outbreak and had a stronger working alliance), would perceive the transition more favorably and experience it as less harmful than those in the initial stages of treatment. We further hypothesized that greater concerns relating to the virus and potential contagion [22] would cause patients to adhere to confinement measures and would therefore be associated with a more positive attitude toward online treatment.

The new measure developed in the current study could be used in future research about obligatory transitions from traditional to online treatment. In addition, identification of the potential factors influencing patient perspectives could guide further research regarding this process of transition and inform our knowledge regarding any future such changes resulting from the ongoing COVID-19 pandemic or other disruptive events.

## Methods

### Participants

Participants included 63 patients recruited from the patient population of the Hadarim Eating Disorders Treatment Center (EDTC) in Kfar Saba, Israel, a part of the Shavata Mental Health Center. Twenty-four (38%) of these patients were diagnosed with anorexia nervosa (AN) (AN: *n* = 17, atypical AN: *n* = 7), 20 (32%) with bulimia nervosa (BN), 16 (25%) with binge eating disorder (BED), and 3 (5%) with other EDs (purging disorder: *n* = 1, avoidant restrictive food intake disorder: *n* = 2). As presented in Table 1, most participants were female (*n* = 57, 91%), their mean age was 27 (SD = 11.47, 27% under the age of 18), and their mean time in treatment was almost one year (M = 317 days, SD = 195). In order to obtain a broad understanding of patients’ perspectives, all patients who had commenced treatment prior to the lockdown were eligible for participation. As the study did not include any intervention, no further inclusion or exclusion criteria were applied.

**Table 1:** Demographic and clinical characteristics (*N* = 63)

**Demographic characteristics**	
Age, years: M (SD), range	27.25 (11.47), 12–56
Gender: n (%)	
Female	57 (90.50)
Male	6 (9.50)
Marital Status: n (%)	
Single	38 (60.32)
Married/living with partner	23 (36.51)
Divorced	2 (3.17)
Other	
Level of Education: n (%)	
Student	17 (27.0)
High school graduate	15 (23.80)
University student	8 (12.70)
University/college graduate	23 (36.50)
**Clinical characteristics**	
Diagnosis: n (%)	
Anorexia nervosa	24 (38.10)
Bulimia nervosa	20 (31.75)
Binge eating disorder	16 (25.40)
Other ED	3 (4.75)
Age of ED onset: M (SD), range	15.39 (4.99), 12–56
BMI (last measured), kg/m^2^: M (SD), range	24.82 (7.40), 16.36–52.61
Duration of treatment, days: M (SD), range	317 (195), 42–909
Past ED hospitalization: n (%), range	14 (22.22)

*Abbreviations*: *M* mean, *SD* standard deviation, *BMI* body mass index, *ED* eating disorder

### Procedure

The institutional review board (IRB) of the Shalvata Mental Health Center approved the study in a special meeting convened to discuss COVID-19 related studies. In terms of social distancing concerns, the IRB required that when informed consent could not be signed in the clinic, it would be obtained via email. In the case of minors, parents signed the informed consent, in addition to which the under-18 participants also signed an assent form. Eating disorder diagnoses were decided in accordance with the DSM-5 and in clinic discussions held among a trained psychiatrist (YDL) with 5 years of experience in the field, a qualified dietician (RGS) with 20 years of experience, and the head of the center (EZ), with over 30 years of experience in the field. Demographic and ED-related information were collected in the survey, and the last measured body mass index (BMI) was collected from the patient files. Approximately 95% of patients in the clinic were invited to participate in the study. Those who were not approached were those for whom there were certain clinical indicators suggesting that participation would have a negative impact, or those who could not be reached during the lockdown. Fifty-one adult patients were contacted of whom 46 completed the survey. Thirty minor patients and their parents were contacted, and 17 of them participated in the study, with about half of the refusals being due to a parental decision. Overall, consent for participation was approximately 80%, with a higher rate in the adult patients.

Data were collected between mid-April and mid-May 2020, following the March–April lockdown in Israel, which was gradually lifted during the month of May. As of the second week of March, all non-urgent services in the Hadarim clinic (including psychotherapy, and dietetic and psychiatric consultations) were switched to online platforms.

### Measures

The Telemedicine Satisfaction Questionnaire (TSQ [23]) was used to evaluate reactions to remote treatment. This validated tool includes 15 items rated on a 1–5 Likert-type scale and comprises three factors including quality of care, similarity of remote meetings to face-to-face meetings, and perception of the interaction. It has been previously used in studies of videoconferencing-psychotherapy [24, 25]. The internal consistency of the TSQ in this study was α = 0.67 for the total score, with α = 0.50 for the similarity to face-to-face encounter subscale and α = 0.63 for the quality of care subscale.

The Eating Disorder Examination Questionnaire (EDE-Q [26]) was used to assess ED symptoms. The EDE-Q has been used extensively in the study of EDs [27], and the Hebrew version has demonstrated good convergent validity [28]. The internal consistency of the EDE-Q in the present study was α = 0.93 for the global score, and α = 0.63, α =0.79 α = 0.75 α = 0.74 for the restraint, eating concerns, shape concerns, and weight concerns subscales, respectively.

The Depression Anxiety and Stress Scales – Version 21 was used to examine general psychopathology (DASS-21 [29]). This instrument has shown high internal consistency and concurrent validity scores [30]. The internal consistency of the DASS-21 in the present study was α = 0.95, with α = 0.92, α =0.84 and α = 0.90 for depression, anxiety, and stress scores, respectively, similar to reliability scores reported in previous studies of the Hebrew version [31].

Working alliance was assessed using the short version of the Working Alliance Inventory (WAI-S [32]), a widely used instrument, the Hebrew version of which has also been used extensively in studies [33]. The internal consistency in the present study was α = 0.82.

Fears and worries related to the COVID-19 pandemic were assessed with an instrument recently and purposively developed: the fear of COVID-19 scale (FCV-19S [34]). The Hebrew version of the questionnaire was found to have good psychometric properties [22]. The internal consistency in the present study was α = 0.84.

Perspectives toward the transition to online treatment were measured using the six items that we developed for this study. As detailed above, we utilized the TSQ [23] to evaluate reactions and technical aspects of remote treatment. However, the TSQ does not address the obligatory change in mode of therapy that was at the heart of our study. As such, we used previous studies to identify central themes related to patients’ perceptions toward telehealth services, including quality of professional care, preference of telehealth vs. traditional face-to-face treatment, possible future use, and promotion of service to others [35, 36]. Based on these emerging themes we composed six statements, focusing on the transition, to be rated on a 1–5 Likert-type scale. The first and second authors generated the items, which were then reviewed in discussions regarding content by all authors – a psychiatrist, two social workers, and a dietician – all of whom have worked clinically with patients with EDs for many years. The items were also reviewed by several patients with EDs.

Statements 1–4 addressed the perception of the care given as either beneficial or adverse. Statement 5 addressed the preference for online vs. face-to-face treatment, and Statement 6 related to the promotion of this mode of therapy to others. A higher score indicated a more favorable attitude towards online treatment, except for items 1 and 2, which were reversed.

1. I feel the transition to online treatment adversely affected the quality of care I receive at the clinic.
2. I feel the transition to online treatment adversely affected the effectiveness of the treatment I receive at the clinic.
3. I feel the transition to online treatment contributed to the quality of care I receive at the clinic.
4. I feel the transition to online treatment contributed to the effectiveness of the treatment I receive at the clinic.
5. If it were possible to continue using online treatment after the COVID-19 pandemic, I would prefer it rather than going back to conventional face-to-face treatment.
6. I would recommend moving to online treatment to my family members and friends.

These six items are not disorder-specific and may be used among a diverse psychiatric patient population (e.g., patients with depression/anxiety). That said, in the current study we utilized this measure only among patients with EDs. The measure is designed to be answered either with paper and pencil or online. Internal consistency of the six items was excellent: Cronbach’s α = 0.878 (internal consistency above 0.7 is considered acceptable for new instruments [37]). For each of the six items, we assessed Cronbach’s α if the item were deleted, and the results ranged from α = 0.866 (Items 1,5) to α = 0.846 (Item 4).

### Data analysis

Data were analyzed using IBM SPSS-25. Results of the exploratory factor analysis (EFA) verified that all six of the items we developed to measure perceptions toward the transition to online treatment belonged to one single factor: Eigenvalue = 3.745 (explaining 62.4% of variance), Kaiser-Meyer-Olkin (KMO) = 0.794 (a value above 0.6 is considered preferred [37]), Bartlett’s Test of Sphericity chi-square (df = 15) = 208.3, *p* < 0.001. Table 2 demonstrates that component loading for each item ranged from 0.848 (Item 4) to 0.735 (Item 1), meaning that no item had to be removed. T-tests and Pearson’s correlations were computed to examine univariate associations of participants’ characteristics with the 6-item measure and the TSQ scales.

**Table 2:** Means and SDs, factor loadings and item total correlations of the 6 items measuring patients’ perspectives towards the transition to online treatment

	Loading	Mean	SD	Item correlation
1. I feel the transition to online treatment adversely affected the quality of care I receive at the clinic.^a^	.735	2.94	1.17	.630
2. I feel the transition to online treatment adversely affected the effectiveness of the treatment I receive at the clinic.^a^	.802	2.97	1.22	.708
3. I feel the transition to online treatment contributed to the quality of care I receive at the clinic.	.805	2.57	1.09	.692
4. I feel the transition to online treatment contributed to the effectiveness of the treatment I receive at the clinic.^a^	.848	2.63	1.08	.753
5. If it were possible to continue using online treatment after the COVID-19 pandemic, I would prefer it rather than going back to conventional face-to-face treatment.	.741	1.95	1.13	.629
6. I would recommend moving to online treatment to my family members and friends.	.803	2.17	1.17	.700

^a^ Items are reversed. *Abbreviations: SD* standard deviation

## Results

### Sample clinical and therapy-related characteristics

Demographic and clinical characteristics are detailed in Table 1. The mean score of the EDE-Q global score was 3.51 (SD = 1.30), comparable with clinical ED samples [38, 39] and above 95% of the general population [40]. Mean scores in the EDE-Q subscales – that is, restraint (M = 3.45, SD = 1.66), eating concerns (M = 2.72, SD = 1.53), shape concerns (M = 4.18, SD = 1.76), and weight concerns (M = 3.69, SD = 1.59) – were also above 95% general population [41].

The DASS-21 depression score was found to be moderate (M = 15.14, SD = 11.27), whereas the anxiety score was mild (M = 8.79, SD = 8.79), similar to the stress score (M = 17.55, SD = 10.60).

Pandemic-related anxiety as measured by the FCV-19S (M = 13.16, SD = 4.89) was slightly lower than what was measured in a larger non-clinical sample in Israel [22].

The therapeutic alliance total score was found to indicate a relatively strong alliance, in the range of previous ED studies (M = 5.32, SD = 1.24 [42, 43];) with similar results in the subscales of task (M = 5.16 (SD-1.51), bond (M = 5.59, SD = 1.24), and goal (M = 5.19, SD = 1.34).

### Perception of online treatment

**Fig. 1:**
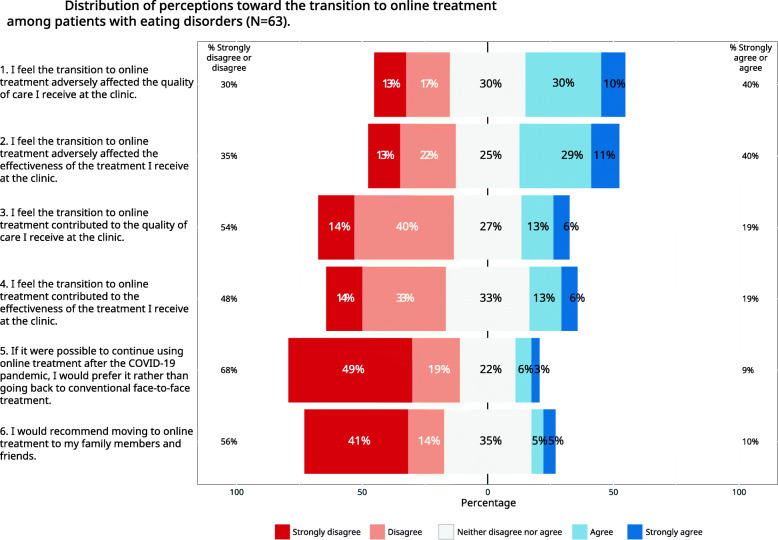
Distribution of perceptions toward the transition to online treatment among patients with eating disorders (*N* = 63)

Figure 1 illustrates participants’ responses to the six Likert-type scale statements on the transition to online treatment. Twenty-five (40%) of the participants agreed that the transition to online treatment adversely affected the effectiveness/quality of their treatment at the clinic, while 22 (34%) disagreed that the effectiveness had been negatively influenced and 19 (30%) disagreed the quality had worsened, leaving the remaining 16 (25%) neutral about the effectiveness and the remaining 19 (30%) neutral about the quality. Twelve (19%) of the participants agreed that the transition to online treatment contributed to the effectiveness/quality of their treatment at the clinic, while 30 (48%) disagreed that the effectiveness had been enhanced and 34 (54%) disagreed that the quality of treatment improved, leaving and the remaining 21 (33%) neutral about the effectiveness and 17 (27%) neutral about the quality.

Most participants (*n* = 43, 68%) stated that they would not prefer continuing online therapy given the choice, with only six participants (9%) opting to continue. Thirty-four participants (54%) stated that they would not recommend online treatment to friends and family members, with 22 (34%) remaining neutral, and only six participants (9%) endorsing it.

The mean score of the TSQ was 3.08 (SD = 0.69), which is lower relative to a mean of around 4.7 in prior reports [24, 25], with subscale means of 3.11 (SD = 0.73) for similarity of online to face-to-face encounter, 3.06 (SD = 0.90) for quality of care provided, and 3.02 (SD = 1.17) for perception of the interaction.

### Univariate associations between demographic, clinical, and treatment-related factors and perceptions of online treatment

Table 3 shows the associations between the Likert-type scale statements and demographic and illness-related characteristics of the participants. Longer duration in treatment was significantly correlated with more positive perceptions towards the transition to online treatment (r = 0.291, *p* = 0.022). Other demographic and clinical characteristics (including BMI and ED diagnosis) were not found to have any significant correlations with the views toward the transition to online therapy.

**Table 3:** The univariate associations of demographic and clinical characteristics with TSQ and the 6-item measure of patients’ perspectives towards the transition to online treatment (*N* = 63)

		TSQ similarity	TSQ quality	Perspectives towards the transition to online treatment
**Demographic characteristics**				
Age	*r =*	.182	−.047	.036
	*p =*	.154	.717	.777
Gender	*r =*	.067	.146	.006
	*p =*	.604	.254	.964
Education	*r =*	.093	−.017	.092
	*p =*	.469	.893	.471
**Clinical characteristics**				
BMI (last measured), Kg/m^2^	*r =*	.221	−.011	.226
	*p =*	.085	.931	.077
Duration of treatment, days	*r =*	.124	−.144	**.291**
	*p =*	.338	.263	**.022**
Past ED hospitalization	*t =*	.149	.061	.152
	*p =*	.242	.634	.235

Note: Perspectives towards the transition to online treatment were measured using the six items developed for the current study. A higher score represents more positive attitude towards the transition. Bold font indicates statistically significant results (*p*-value< 0.05). *Abbreviations*: *TSQ* Telemedicine Satisfaction Questionnaire, *BMI* body mass index, *ED* eating disorder

Table 4 shows the associations between the perceptions and clinical and treatment-related factors. Strength of therapeutic alliance (as measured by the WAI-S total score) was significantly correlated with more favorable perspectives towards the transition to online treatment (r = 0.293, *p* = 0.028). Goal and task scores also correlated with favorable perspectives towards the transition (r = 0.295, *p* = 0.027; r = 0.346, *p* = 0.00, respectively). Fear of COVID-19 significantly correlated with favorable perspectives towards the transition (r = 0.276, *p* = 0.029).

**Table 4:** The univariate associations of eating disorder (ED) psychopathology, general psychopathology, therapeutic alliance, COVID-19 anxiety with TSQ and the 6-item measure of patients’ perspectives towards the transition to online treatment (*N* = 63)

		TSQ similarity	TSQ quality	Perspectives towards the transition to online treatment
**ED psychopathology (EDE-Q)**				
Dietary restraints	*r =*	−.096	.101	−.121
	*p =*	.454	.432	.347
Eating concerns	*r =*	.045	.041	−.094
	*p =*	.726	.751	.466
Shape concerns	*r =*	−.070	−.100	−.168
	*p =*	.587	.435	.187
Weight concerns	*r =*	−.038	−.053	−.155
	*p =*	.767	.678	.224
**General psychopathology (DASS-21)**				
Depression	*r =*	−.121	.094	−.162
	*p =*	.344	.466	.205
Stress	*r =*	−.037	.094	−.158
	*p =*	.772	.461	.215
Anxiety	*r =*	−.114	.026	−.080
	*p =*	.376	.840	.531
**COVID-19 anxiety (FCV-19S)**				
	*r =*	−.193	−.143	**.276**
	*p =*	.129	.263	**.029**
**Therapeutic alliance (WAI-S)**				
Total	*r =*	.196	.173	**.293**
	*p =*	.147	.203	**.028**
Task	*r =*	.033	.086	**.346**
	*p =*	.806	.530	**.009**
Bond	*r =*	**.291**	.119	.140
	*p =*	**.030**	.384	.302
Goal	*r =*	.204	**.268**	**.295**
	*p =*	.131	**.045**	**.027**

Note: Perspectives towards the transition to online treatment were measured using the six items developed for the current study. A higher score represents a more positive attitude towards the transition. Bold font indicates statistically significant results (p-value< 0.05). *Abbreviations*: *ED* eating disorder, *EDE-Q* Eating Disorder Examination Questionnaire, *DASS-21* Depression, Anxiety and Stress Scale – Version 21, *WAI-S* Working Alliance Inventory – short version, *COVID-19* coronavirus disease 2019, *FCV-19S* Fear of COVID-19 Scale (FCV-19S), *TSQ* Telemedicine Satisfaction Questionnaire

The TSQ was found to be correlated only with the therapeutic alliance bond score (similarity: r = 0.291, *p* = 0.030), and goal score (quality: r = 0.268, *p* = 0.045). We also examined whether patients with different ED diagnoses (AN, BN, BED) had different perspectives toward the transition and/or different TSQ scores, and we found no significant results.

## Discussion

The aim of the current study was to conduct a pilot investigation into the perspectives of ambulatory patients with EDs toward the transition from conventional face-to-face treatment to online treatment during the COVID-19 lockdown and to provide a measure for their assessment. Analyses demonstrated three main findings. First, preliminary analyses suggested that these views can be measured using a Likert-type 6-item scale. Second, a mixed distribution of views was observed in our sample with regard to the transition to online treatment. Third, the key factors that were linked with a more favorable attitude toward the transition were longer duration of treatment, stronger therapeutic alliance and stronger COVID-19 anxiety. These findings highlight the need to identify patients who may face difficulties with transitioning to online therapy as well as offering them appropriate support or a service package to meet their needs.

Our preliminary analyses revealed that the six items we developed could provide an accurate measure evaluating patients’ perspectives toward the transition to online treatment. The results of the exploratory factor analysis (EFA) revealed a unidimensional factor structure which explained 62.4% of the variance. Cronbach’s alpha for the unidimensional model was excellent (α =0.878). An examination of the construct validity indicated that the scale was not related to telemedicine satisfaction (as measured by the TSQ), suggesting that the construct measuring patients’ perspectives toward the transition to online treatment should be studied in parallel with the construct measuring patients’ satisfaction with online treatment. Of note is that a prior study analyzing the impact of the lockdown on patients with EDs and obesity did not distinguish between these two constucts [4].

A roughly normal distribution of perspectives was found toward the positive contribution or adverse effect of the transition: an expression of varied perspectives on the transition. Interestingly, although most participants did not report an adverse effect of their online care (Items 1,2), as many as two thirds stated that they would not choose to continue with online treatment (Item 5), and more than half were sure that they would not recommend online treatment to family members and friends (Item 6). This finding suggests that patients understand remote online treatment to be a situation-specific necessity rather than a choice, a finding further supported by the association we found between the goal component of the therapeutic alliance and satisfaction with the quality of telemedicine. Indeed, this transition was previously described by patients with AN as being accompanied by feelings of great uncertainty and a lack of preparedness [44]. Along the same lines, we found lower satisfaction scores across quality, similarity, and perception of encounter measures, compared with previous studies that used the same tool to investigate satisfaction of online treatment for mental health issues in unrelated circumstances [24, 25]. Online treatment in our study was closely connected with the COVID-19 pandemic, an overall negative and anxiety-provoking experience for patients with EDs [45], implicated in the worsening of ED symptoms for many patients [8, 46, 47], and prompting the strong wish to “go back to normal.” Our results contrast with those from a recent report by Fernández-Aranda et al. where a newly developed comprehensive tool to assess the impact of confinement on ED symptoms and acceptance of telemedicine described telemedicine as well accepted [4]. However, the novelty of the tool used in that study precludes comparison to other studies, and the data were collected retrospectively. Our finding that most participants would not willingly choose online treatment is important, as recent publications have called on the professional world to “seize the opportunity” and embrace online therapy, not as a requirement but as an indicated and preferred treatment [48]. Patients may be reluctant to make this transition, and it is important that their views be considered.

With respect to potential contributing factors, no demographic characteristics, including age, were found to be correlated with views on the transition. As hypothesized, longer pre-lockdown duration and a stronger therapeutic alliance were found to be associated with a more positive view of the transition. A meta-analysis comparing videoconferencing-psychotherapy (VCP) to in-person therapy found the working alliance to be good in VCP, but inferior in relation to face-to-face therapy, although symptom reduction was comparable in both groups [49]. Given the challenge of establishing an early alliance remotely [7], it could be that participants who did not have a chance to develop a strong therapeutic alliance before the lockdown (e.g., due to short treatment duration or preexisting tensions with the therapist) had difficulties strengthening their alliance during the lockdown in the context of VCP usage. This lack of alliance may have thus contributed to their negative perception of online treatment. These findings stress the importance of the therapeutic alliance as a central component of therapy [50], which in this context likely facilitated (or impeded, where there was no such alliance) the transition from the patient’s perspective. Our findings mirror the recent experience as described by clinicians; namely, previous face-to-face engagement with a therapist was found to allow for a smoother transition to distant ED protocol-based treatment during the pandemic, whereas initiating the program distantly was greeted by difficulty in building a collaborative relationship [51].

Clinical factors including severity of ED symptoms, depression, or general anxiety showed no overall effect on perceptions toward the transition. Patients with different ED diagnoses (AN, BN, or BED) did not vary in their satisfaction with online treatment or in their perceptions towards the transition, a finding that may be attributed to the transdiagnostic view of EDs [52], which focuses on the common features, rather on the dissimilarities, of ED diagnoses [53]. This finding differs from that described by Fernández-Aranda et al. who found lower acceptance of online therapy among AN patient. Future studies using larger samples with more patients in each diagnostic group would be helpful in clarifying these conflicting results.

As predicted, fear of the novel coronavirus and the disease that it causes was associated with positive views toward online treatment. Concerns among people with EDs regarding the impact of COVID-19 on their health and mental health have been found to be common [45]. For health-anxious patients, online therapy was advantageous for two reasons. First, their fear would have prevented them from leaving home and arriving at clinics for therapy anyway; as such, remote treatment enabled them to continue care. Second, as fears of COVID-19 have indeed been found to predict mental health symptoms [54] (e.g. depression, anxiety), therapy could potentially provide a containing environment to ease these fears and alleviate their possible harmful results.

The strengths of our study include the use of validated questionnaires as well as a dedicated measure, developed specifically for this study, and conducting a rapid real-world data collection among a clinical sample during the lockdown period, thus capturing patients’ perceptions during this unique and unprecedented time. Some important limitations should be noted. Our sample size was limited by the size of our center and the short time in which the study was conducted. In our study we grouped together patients with different ED diagnoses, an approach which is supported by the ED transdiagnostic model [53]. Future studies using a larger sample size may uncover differences among the main ED diagnoses. Such studies could also expose variance between attitudes of patients undergoing different models of therapy, for example enhanced cognitive behavioral therapy (CBT-E) against insight-directed therapy. Given the exploratory nature of our study, alpha correction for multiple comparisons was not used in the analyses. Future studies may verify our results using a larger sample size which would allow alpha correction. The design of the study was cross-sectional, and therefore causal relations cannot be deduced from our findings. Last, it could be that our clinic has specific characteristics (e.g., a large portion of chronic patients with a relatively long treatment duration) that may have impacted results, thus limiting the generalizability of our findings to other clinical ED populations.

## Conclusions

Our pilot study demonstrates that the views of ED outpatients can be assessed using a short 6-item Likert-type scale. Our main preliminary conclusions are that a longer pre-lockdown treatment duration, stronger therapeutic alliance, and more severe COVID-19 anxiety are associated with a positive attitude toward online treatment. These findings require further investigation in larger samples, potentially via the utilization of our transition-focused measure. Amidst the ongoing pandemic situation and confinement measures that may be instituted on a not infrequent basis, initial clinical implications drawn from our findings are that special consideration may be needed in newly-begun treatments, and that attentiveness and a flexible approach should be taken towards those prone to health anxieties. Future studies might wish to utilize a prospective longitudinal design in order to determine how views on the transition are related to long-term treatment outcomes.

## Data Availability

Data generated or analyzed in the study are available from the authors upon reasonable request.

## Abbreviations

AN: Anorexia Nervosa
BED: Binge Eating Disorder
BMI: body mass index
BN: Bulimia Nervosa
CBT-E: Enhanced Cognitive Behavioral Therapy
COVID-19: Coronavirus disease of 2019
DASS-21: Depression Anxiety and Stress Scales
ED: Eating disorder
EDE-Q: Eating Disorder Examination Questionnaire
EFA: Exploratory Factor Analysis
FCV-19S: Fear of COVID-19 Scale
IRB: Institutional Review Board
KMO: Kaiser-Meyer-Olkin
SARS-CoV-2: Severe Acute Respiratory Syndrome Coronavirus 2
TSQ: Telemedicine Satisfaction Questionnaire
VCP: Video-Conferencing Psychotherapy
WAI-S: Working Alliance Inventory
